# Comparative Assessment of Tubular Ceramic, Spiral Wound, and Hollow Fiber Membrane Microfiltration Module Systems for Milk Protein Fractionation

**DOI:** 10.3390/foods10040692

**Published:** 2021-03-24

**Authors:** Roland Schopf, Florian Schmidt, Johanna Linner, Ulrich Kulozik

**Affiliations:** Chair of Food and Bioprocess Engineering, TUM School of Life Sciences, Technical University of Munich, Weihenstephaner Berg 1, 85354 Freising, Germany; florian.schmidt@uni-hohenheim.de (F.S.); johanna.linner@gmx.de (J.L.); ulrich.kulozik@tum.de (U.K.)

**Keywords:** separation efficiency, casein, whey protein, deposit layer formation, fouling, transmembrane pressure, limiting flux, transmission, mass flow

## Abstract

The fractionation efficiency of hollow fiber membranes (HFM) for milk protein fractionation was compared to ceramic tubular membranes (CTM) and spiral wound membranes (SWM). HFM combine the features of high membrane packing density of SWM and the more defined flow conditions and better control of membrane fouling in the open flow channel cross-sections of CTM. The aim was to comparatively analyze the effect of variations in local pressure and flow conditions while using single industrially sized standard modules with similar dimensions and module footprints (module diameter and length). The comparative assessment with varied transmembrane pressure was first applied for a constant feed volume flow rate of 20 m^3^ h^−1^ and, secondly, with the same axial pressure drop along the modules of 1.3 bar m^−1^, similar to commonly applied crossflow velocity and wall shear stress conditions at the industrial level. Flux, transmission factor of proteins (whey proteins and serum caseins), and specific protein mass flow per area membrane and per volume of module installed were determined as the evaluation criteria. The casein-to-whey protein ratios were calculated as a measure for protein fractionation effect. Results obtained show that HFM, which so far are under-represented as standard module types in industrial dairy applications, appear to be a competitive alternative to SWM and CTM for milk protein fractionation.

## 1. Introduction

The fractionation of skim milk into micellar casein and whey proteins by means of microfiltration (MF) is an established unit operation in the dairy industry. Ceramic tubular membranes (CTM) [1,2,3,4,5,6,7,8] or spiral wound membranes (SWM) [9,10,11,12] are usually used for this purpose. Whereas SWM provide a high packing density, i.e., high membrane area per module [11], CTM are more resistant, more durable, and they are usually reported to have better separation characteristics [1,3]. However, CTM are expensive [13] and SWM more difficult to clean [14]. Polymeric hollow fiber membranes (HFM) generally combine the advantages of both types of modules, including a large membrane area per module, free flow cross-sections, low cost, and high separation efficiency [13]. Until recently, HFM were rarely used in the dairy sector, although they have been the standard technique in other fields of food processing, like drinking water production, wastewater treatment, and wine and beer filtration [15].

The deposit formation of retained matter is an issue in all membrane systems, as reported for HFM [15,16,17,18,19], CTM [1,6,7], and SWM [9,11]; however, at different levels due to the different flow channel configurations. Deposit formation strongly impairs flux and the desired high whey protein mass flow, which is a key aspect in milk protein fractionation [7]. Increased transmembrane pressure, ∆*p_TM_*, leads to greater transport towards the membrane and results in the compaction of the layer of retained casein micelles deposited on the membrane surface, despite crossflow conditions [20,21,22,23,24,25], thereby reducing the porosity of the deposited layer. This creates additional steric resistance to the mass transport of whey proteins and, thus, results in a reduction of whey protein transmission [11]. Bouchoux et al. [25] showed that casein micelles up to concentrations of 125 g·L^−1^ do not significantly interact with one another. However, the casein deposit layer becomes more compact, due to the dewatering of the micelles and a gel formation at higher concentrations of about 200 g·L^−1^. Qu et al. [24] reported a critical osmotic pressure of 0.35 bar to be sufficient for irreversible gel formation on the membrane surface. In CTM, the volume flow and resulting wall shear stress can generally be increased without limitation to reduce deposit layer formation by higher wall shear stress [1]. However, in SWM, the increase in feed volume flow is limited due to structural module instability, occurring above critical backpressure levels [14,26] which results in module damage, e.g., by the so-called telescoping effect [27]. For milk protein fractionation, flux and whey protein transmission are the most important factors. Next to that, the casein-to-whey protein ratio as the main fractionation effect is an additional decisive evaluation aspect when assessing module types in this context. A deposit-induced reduction of whey protein transmission is the more decisive criteria in the case of milk protein fractionation than flux reduction, as flux reduction can be compensated by increasing the installed membrane area. Given the module characteristics of HFM, the question was whether the less intense deposit formation in open tubular membrane systems, like in CTM, can be beneficially combined with the advantage of HFM, namely high membrane area packing density, which is also a key feature of SWM.

In previous studies, we have reported on MF-based milk protein fractionation by HFM [16,17,18,19], however with another perspective on fundamental aspects of deposit formation; therefore, small lab scale modules have been utilized. We also demonstrated that the length dependency in HFM is equal to that of CTM, and that the length-dependent pressure drop is responsible for inhomogeneous fouling along the HFM [19]. Data for milk protein fractionation using industrially-sized HFM modules are still lacking. Various works reported on milk protein fractionation using different module systems or only comparing SWM with CTM, excluding HFM. Adams and Barbano [28] compared the filtration performance of ceramic membranes with different channel diameters, but the same outer module diameter. They showed that ceramic membranes with 6 mm channel diameters could achieve a higher limiting retentate protein concentration than membranes with a channel diameter of 4 mm at the same pressure drop and the same flux due to an increase in crossflow velocity. Moreover, there are some concepts for counteracting the length-dependent effects and obtaining a more homogenous flux and deposit layer formation. In the so-called uniform transmembrane pressure (UTP) mode, ∆*p_TM_* is kept constant by recirculating the permeate to a permeate side pressure drop [29,30]. The disadvantage of this concept is the higher energy demand and additional investment costs. Another option is to integrate an additional resistance to permeation from inlet to module outlet, either by the variation of the thickness of the selective layer or integrating a gradient in membrane resistance in the support layer [31].

To our knowledge, only one study has compared the filtration performance of skim milk MF with different filtration modules [10]. Zulewska et al. [10] revealed significant differences in the flux of SWM (16 kg·h^−1^·m^−2^) and ceramic gradient membrane (72 kg·h^−1^·m^−2^) at a filtration temperature of 50 °C, but HFM were not investigated. The authors found a higher β-lactoglobulin passage through the CTM as compared to SWM, attributing this to the material properties of the ceramic membrane [10]. The effects of different ∆*p_TM_* on the formation of the deposit layer were not included in their assessment. Overall, it can be stated that the conditions were such that results from the various studies were not obtained from comparable experimental set-ups, i.e., using different processing conditions, differently sized module types, and different evaluation criteria.

Because the module systems at the industrial level are operated under different volume throughput conditions to achieve the respective desired maximally possible fluid velocities and wall shear stresses for each system, a question for this study was how to compare HFM, CTM, and SWM in this regard. The options were to operate the systems at either constant pressure drop or at constant feed volume flow. Therefore, the comparative assessment was performed applying both concepts to realize a relevant comparison of the membranes at common and approximately optimal crossflow velocity and wall shear stress conditions or at the same feed input. The volume flow and the pressure drop that were chosen were those determined as the maximum for SWM, given the risk of module destruction by membrane telescoping at higher backpressures.

A key aspect and aim were to compare all three membrane systems at their whey protein mass flow optimum in order to produce results that were closer to industrial reality using similarly sized commercial modules with similar geometric dimensions, i.e., module footprints, and to use the module performance as the assessment criteria. This enables a comparison of flux, transmission, and whey protein mass flow in relation to the individual module unit rather than per square meter installed membrane area. A comparably systematic approach has not been reported so far to our knowledge.

The expectation was that HFM should perform well when compared to CTM in terms of specific flux in L m^−2^ h^−1^ and superior to SWM in terms of flux and mass flow per module volume. This should lead to a higher impact on flux and transmission of HFM, resulting in a higher fractionation efficiency when compared to CTM and SWM.

## 2. Materials and Methods

### 2.1. Milk

Pasteurized skim milk (74 °C for 28 s) was sourced from a local dairy (Molkerei Weihenstephan, Freising, Germany) and stored (4 °C) for up to five days. The milk pH was 6.6 for all experiments. Table 1 shows the viscosity, *η*, and density, *φ*, of deionized water, skim milk, and the microfiltration permeate measured according to Schopf et al. [19].

### 2.2. Membrane Modules

The nominal pore size of the membranes was 0.1 µm according to manufacturer specifications. HFM (polyether sulfone, inner fiber diameter 1.5 mm, outer fiber diameter 2.25 mm, and filtration from inside-out) were supplied by Pentair-X-Flow (Enschede, The Netherlands) for an industrial module (R100MF, 1932 fibers) and a self-made module with ten fibers to gain higher crossflow velocities. SWM (V01, module configuration 6338, polyvinylidene fluoride, equipped with a 46-mil diamond spacer) were supplied by Synder Filtration (Vacaville, CA, USA) with two cartwheel-shaped anti-telescoping devices. CTM (7P19-40, seven elements each with 19 channels, selective layer material α-Al_2_O_3_, a diameter of each channel 4.0 mm) were supplied by Pall Corporation (Port Washington, NY, USA). The three industrial module types in their dimensions with approximately roughly the same module diameters and membrane lengths were chosen to perform a meaningful comparative assessment of the modules (Table 2).

### 2.3. Conditioning and Cleaning Procedure

At the beginning of the filtration, a conditioning procedure was carried out. At 55 °C polymer HFM and polymer SWM were conditioned for 20 min. with 0.4% *v/v* Ultrasil 69 (Ecolab Deutschland GmbH, Monheim/Rhein, Germany). Ceramic CTM were conditioned for 20 min. with a caustic agent (0.5% UF466, Halag Chemie AG, Aadorf, Switzerland) at 65 °C. Subsequently, membranes and filtration plants were rinsed (10 min) with deionized water. At the end of the filtration, 0.8% *v/v* Ultrasil 69 and 0.3% *v/v* Ultrasil 67 were used for 40 min. cleaning procedure (55 °C) for the polymer HFM or polymer SWM. Subsequently, 0.4% *v/v* Ultrasil 75 was used at 55 °C for 30 min. For ceramic CTM 1% UF466 at 65 °C for 40 min, followed by acidic cleaning (0.5% nitric acid (60%), Halag Chemie AG, Aadorf, Switzerland) at 65 °C for 20 min. The pure water flux was measured to ensure that the membranes were clean before filtration.

### 2.4. Filtration Plant

The pilot plant (SIMA-tec GmbH, Schwalmtal, Germany) was had with a multistage centrifugal pump (CRNE, Grundfos GmbH, Erkrath, Germany). The feed volume flow and the permeate volume flow were measured with electromagnetic flowmeters (FEH311, Endress + Hausser GmbH+Co. KG, Weil am Rhein, Germany). Digital pressure transmitters (A-10, WIKA Alexander Wiegand SE & Co. KG, Klingenberg, Germany) detected the pressure before and after the membrane and in the permeate. A digital resistance thermometer measured the temperature (TR30, WIKA Alexander Wiegand SE & Co. KG, Klingenberg, Germany). The filtration was recorded using ServiceLab 12 (ServiceLab, Neu-Ulm, Germany).

### 2.5. Filtration Conditions

The microfiltration unit was tempered with pure water at 55 °C prior to filtration to avoid initial temperature changes affecting the filtration process. The water flux was recorded at a feed volume flow of 15 m^3^·h^−1^ at a temperature of 55 °C and ∆*p_TM_* of 0.1, 0.2, 0.3, 0.4, and 0.5 bar. Separately, the skim milk was tempered at 55 °C before being transferred to the feed tank. Because of the lower viscosity of the retentate and the permeate leading to higher flux when compared to cold temperatures, the filtration temperature of 55 °C was chosen. Moreover, this temperature is above the optimum for microbial growth in milk [6] and below the denaturation temperature of the milk proteins [32]. Starting the filtration, the remaining water was trained with skim milk, while the permeate valve was closed. The mixed-phase was drained. Afterward, the retentate was recirculated into the reservoir. Two filtration protocols were performed, which were applied independently of each other. First, the fractionation was carried out with the industrial membrane modules at a constant feed volume flow of 20 m^3^·h^−1^ for a better comparison of the membranes at the same feed input. Second, the fractionation was carried out at a constant pressure drop of 1.3 bar·m^−1^ for a better comparison of the membranes at common crossflow velocity and wall shear stress conditions. The reason for choosing this feed volume flow and pressure drop can be found in the limitation of SWM by structural damages at a higher pressure drop, as discussed in the results section in detail. A self-made module with ten fibers was used for this propose to ensure high pressure drop and crossflow velocity in the hollow fiber. The mean ∆*p_TM_* variations for both approaches were done by a stepwise increase and ∆*p_TM_* was adjusted to 0.5 bar by opening permeate valve. The permeate mixed-phase was drained and the permeate was recirculated into the feed tank. ∆*p_TM_* was held constant for 45 min. to achieve steady-state filtration conditions throughout. Subsequently, ∆*p_TM_* was gradually increased (every 30 min.) to 0.75, 1.00, 1.25, 1.50, and 2.00 bar to obtain filtration data under the steady-state condition for each step.

### 2.6. Analysis of Caseins and Whey Proteins

Permeate and retentate were quantitatively analyzed in the amount and composition of their milk proteins via reversed-phase high-performance liquid chromatography (RP-HPLC), according to Dumpler et al. [32].

### 2.7. Calculations

The transmembrane pressure, ∆*p_TM_*, was determined according to Equation (1):

(1) $${\Delta p}_{TM}=\frac{p_{inlet}+p_{outlet}}{2}-p_{permeate}$$

The pressure drop, ∆*p_L_*, was calculated as the pressure at the inlet, *p_inlet_*, minus the pressure at the membrane outlet, *p_outlet_* (Equation (2)):

(2) $$\Delta p_{L}=p_{inlet}-p_{outlet}=\frac{\tau_{W}\cdot d_{i}}{4\cdot L}$$

where *τ_W_* is the shear stress, *d_i_* is the tube diameter, and *L* the length of the tubular membrane. For a comparison of the wall shear stress, *τ_W_*, the wall shear rate, *γ_T_*, was also calculated according to Newton’s law of viscosity, *η*, (Equation (3)) assuming the fluid to be Newtonian at the membrane surface [33,34]:

(3) $$\gamma_{T}=\frac{\tau_{W}}{\eta }$$

For tubular flow, the pressure drop, ∆*p_L_*, is a function of the crossflow velocity, *v*, (Equation (4)):

(4) $$\Delta p_{L}=\frac{1}{2}\cdot \lambda \cdot \frac{L}{d_{i}}\cdot \rho \cdot v^{2}$$

with the friction factor, *λ*, and the density, *ρ*. Therefore, the velocity, *v*, is the ratio of the feed volume flow, ${\dot{V}}_{feed}$, and the area of the cross-section, *A_sec_*, (Equation (5)):

(5) $$v=\frac{{\dot{V}}_{feed}}{A_{sec}\;}$$

The installed membrane area per module, i.e., the so-called packing density *Θ*, was calculated as a ratio of the area of the membrane *A_Membrane_* and the module volume *V_Module_* (Equation (6)):

(6) $$\Theta =\frac{A_{Membrane}}{V_{Module}\;}$$

The flux *J* was calculated according to Darcy’s law (Equation (7)). The sum of the membrane resistance, *R_M_*, plus the deposit layer resistance, *R_D_*, was the filtration resistance, *R_F_*:

(7) $$J=\frac{{\dot{V}}_{per}}{A_{Membrane}}=\frac{\Delta p_{TM}}{\eta \cdot R_{F}}=\frac{\Delta p_{TM}}{\eta \cdot \left(R_{M}+R_{D}\right)}$$

The protein transmission, $Tr_{\mathrm{WP}}$, (sometimes also referred to as ‘permeation’ in literature) was determined as (Equation (8)):

(8) $$Tr_{\mathrm{WP}}\;=100\%\cdot \frac{c_{\mathrm{WP},\mathrm{per}}}{c_{\mathrm{WP},\mathrm{ret}}}$$

where *c*_i,per_ was the permeate concentration and *c*_i,ret_ the retentate concentration. The whey protein mass flow, ${\dot{m}}_{\mathrm{WP}}$, was determined as (Equation (9)):

(9) $${\dot{m}}_{\mathrm{WP}}=J\cdot c_{\mathrm{WP},\mathrm{per}}$$

### 2.8. Statistics

Origin 2020b (Origin Lab Corporation, Northampton, MA, United States) was used to plot the graphs. Statistical analysis was done with RStudio 1.3.1093 (RStudio, Boston, MA, USA). The *p*-value that was given from a one-way analysis of variance was calculated to indicate the significance level. The data points in the graphs represent the average and the error bars represent the range of the standard deviation of two filtrations.

## 3. Results and Discussion

### 3.1. Influence of the Module Configuration on the Flux

**Figure 1:**
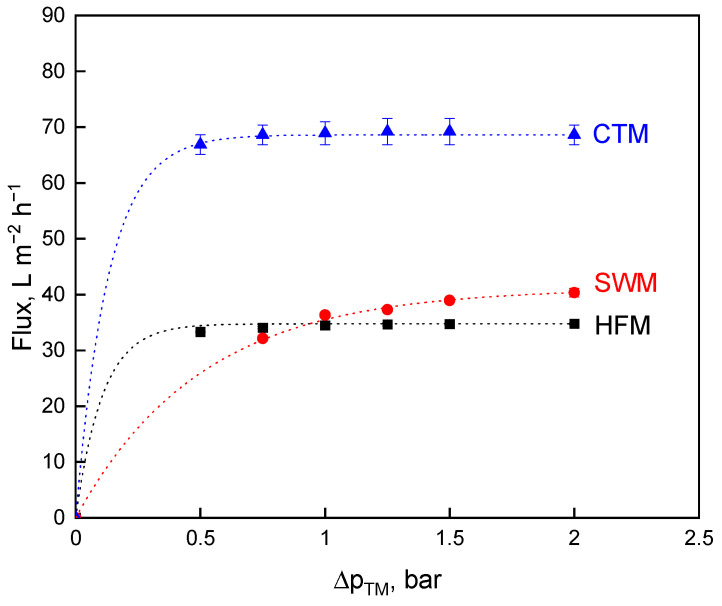
Flux as a function of the transmembrane pressure, ∆*p_TM_*, for spiral wound membrane (SWM; •), ceramic tubular membrane (CTM; ▲), and hollow fiber membrane (HFM; ■) at a feed volume flow of $\dot{V}$ = 20 m^3^·h^−1^.

The permeate flux was used as the first criterion for investigating the influence of the transmembrane pressure, ∆*p_TM_*, variation on the deposit layer formation and, thus, on the efficiency of milk protein fractionation. High flux values can be achieved by high pressure drops due to increased deposit removal. SWM had high frictional effects, due to the module design with spacer geometry on the retentate side [26]. In contrast, CTM and HFM have free flow channel cross-sections [19]. For this reason, the pressure drop, ∆*p_L_*, in CTM and HTM is lower than in SWM. The maximum permissible ∆*p_L_* of 1.3 bar·m^−1^ [35] to prevent structural damage in the SWM was reached at a mean crossflow velocity, *v*, of 0.6 m·s^−1^ and a feed volume flow rate, $\dot{V}$, of 20 m^3^ h^−1^. This feed volume flow rate was also first applied for HFM and CTM as one of the options of parameter choice. Figure 1 depicts the measured flux values.

Figure 1 shows the typical relationship for flux over the ∆*p_TM_* for HFM [19], SWM [11], and CTM [6]. Increasing ∆*p_TM_* leads to a flux increase before the curve starts to level off (critical flux level), then approaching an upper level referred to as limiting flux [36]. Intense membrane fouling occurs at higher ∆*p_TM_* levels. The amount of protein that is deposited on the membrane surface increases and the deposited layer gets gradually more compressed [22]. In particular, casein micelles forming the deposited layer will be compressed into a gel-like structure that reduces flux and permeability [20,24]. The critical flux was reached at different ∆*p_TM_* levels, depending on the membrane module. At a ∆*p_TM_* of 2.0 bar, the highest flux value could be achieved with CTM (67 L m^−2^·h^−1^), followed by SWM (41 L m^−2^·h^−1^) and HFM (35 L·m^−2^·h^−1^). The flux values of SWM and HFM did not significantly (*p* ≤ 0.1) differ from each other at all ∆*p_TM_* levels. Polymeric membranes have a higher hydrophobicity when compared to ceramic membranes [37], thus the flux of CTM is significantly (*p* ≤ 0.001) higher than that of HFM and SWM.

The comparison via constant feed volume flow is not the perfect choice of conditions, because the modules are operated differently at the same volume flow in terms of pressure drop, wall shear stress, *τ_w_*, and crossflow velocity, *v*. Except for SWM, the modules were thus operated at volume flow rates, and resulting wall shear stresses and crossflow velocity levels were lower than under industrial conditions, but still in a range of practical relevance (Table 3).

In the case of SWM, the velocity (*v* = 0.6 m·s^−1^) is in the upper range (maximum permissible pressure drop of 1.3 bar·m^−1^) of commonly applied crossflow velocities [11]. Hartinger et al. [35] described that flux and whey protein mass flow in SWM increase with an increasing crossflow velocity until a maximum at mean crossflow velocities of 0.6–0.78 m·s^−1^ is reached. Typical crossflow velocities for HFM are 0.5–5.8 m·s^−1^ [18,19]. For CTM, 3.3 m·s^−1^ (*τ_w_* = 76 Pa) is also a common crossflow velocity for milk MF, but rather low: Samuelson et. al. [1] mentioned 1.5 m·s^−1^ to a maximum of 8 m·s^−1^ as the typical range of crossflow velocities in ceramics membranes. Moreover, Schiffer et al. [38] reported the optimum for limiting flux at wall shear stresses of 131 Pa. The authors observed that higher wall shear stresses lead to flux reduction. Thus, too high velocities could not only reduce flux, but also reduce the minimal adjustable transmembrane pressure of a module, where no backpressure is builds up [19]. Furthermore, focusing on the wall shear rate, as calculated from Equation (3), the values of 82,000 s^−1^ for CTM and 25,000 s^−1^ for HFM seems to be rather high, but it is, as reported by Gésan-Guiziou et al. [33] for CTM, in a common range. They calculated wall shear rates up to 168,000 s^−1^. However, they also described that the wall shear stress is a more suitable value for describing the tubular flow properties on deposit layer formation as compared to the wall shear rate. Therefore, the wall shear stress and the corresponding pressure drop are used for the description of the influence of the module configuration on the flux.

When comparing the membranes with a constant volume flow, HFM and CTM are supposedly disadvantaged due to the crossflow velocity below industrial standard. Therefore, HFM (*τ_w_* = 48 Pa, *v* = 3.2 m·s^−1^) and CTM (*τ_w_* = 127 Pa, *v* = 4.7 m·s^−1^) were also operated at the same pressure drop of 1.3 bar·m^−1^, like the SWM, at common industrial conditions and approximately at their optimums, as discussed above (Table 4).

**Figure 2:**
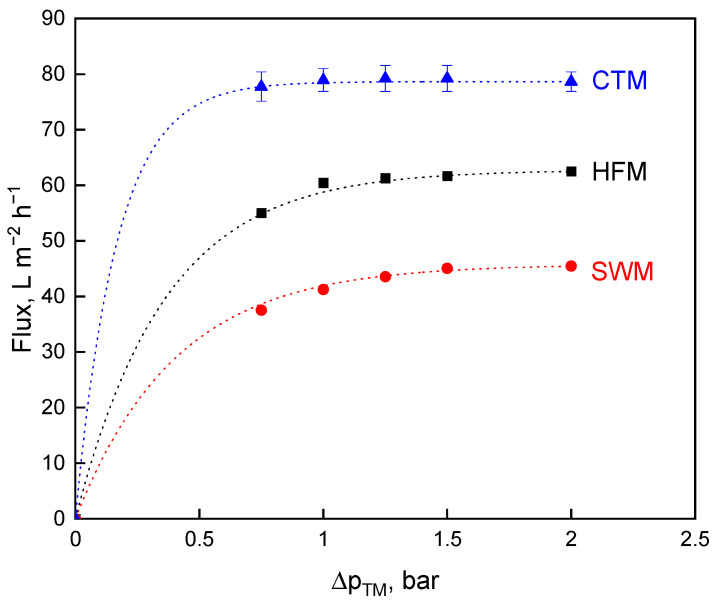
Flux as a function of the transmembrane pressure, ∆*p_TM_,* for spiral wound membrane (SWM; •), ceramic tubular membrane (CTM; ▲), and hollow fiber membrane (HFM; ■) at a pressure drop of ∆*p_L_* = 1.3 bar·m^−1^.

The flux values for all three membrane types as a function of the ∆*p_TM_* were given in Figure 2 at a constant pressure drop of 1.3 bar·m^−1^.

Figure 2 demonstrates that higher flux values could be reached with higher wall shear stress for CTM and HFM as compared to the results shown in Figure 1. High crossflow velocities lead to an increase in the eroding forces removing or at least mitigating the effect of deposit layer formation. Operating at approximately their crossflow velocity optima, there are differences in the performance of the membranes operated at the same volume feed flow rate. When comparing the three membranes in terms of flux, CTM indicate the highest limiting flux at 79 L·m^−2^·h^−1^, followed by HFM (62 L·m^−2^·h^−1^) and SWM (45 L·m^−2^·h^−1^). The flux of CTM, HFM, and SWM differed significantly (*p* ≤ 0.001) from each other. The flux values of CTM and SWM are slightly higher than that reported by Zulewska et al. [10] due to the higher pressure drop. Ceramic membranes are more hydrophilic as compared to polymeric membranes, resulting in lower protein adsorption to the membrane [37]. This effect might well play a role, but we postulate that the flow properties in SWM with irregular flow velocity profiles before and behind the spacer net filaments were mainly responsible for the faster and more locally intense deposit layer formation producing a higher deposit layer resistance as compared to the tube flow in HFM.

### 3.2. Influence of the Module Configuration on Milk Protein Transmission

**Figure 3:**
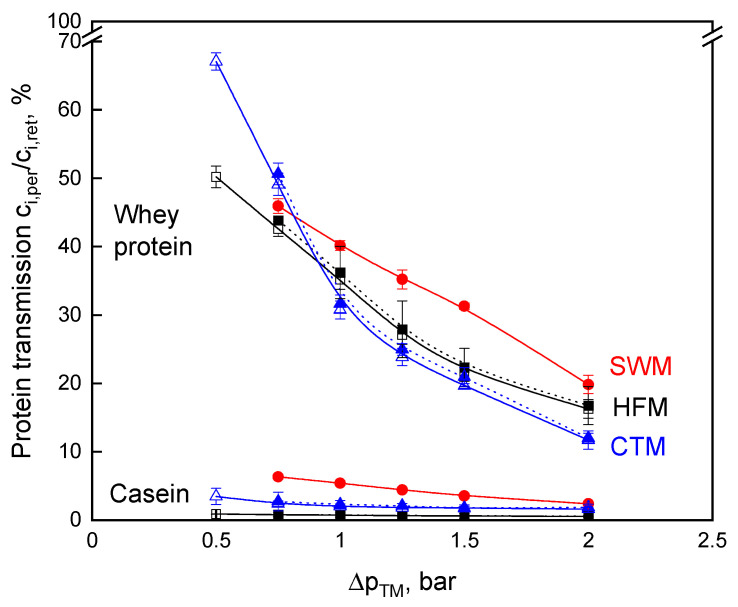
Protein transmission as a function of transmembrane pressure, ∆*p_TM_*, for skim milk microfiltration for spiral wound membrane (SWM; •), ceramic tubular membrane (CTM; △ *τ_w_* = 76 Pa; ▲ *τ_w_* = 127 Pa), and hollow fiber membrane (HFM; □ *τ_w_* = 23 Pa; ■ *τ_w_* = 48 Pa).

The permeate protein composition and protein amount at different ∆*p_TM_* was analyzed to assess the influence of ∆*p_TM_* variation on the transmission of milk proteins (Figure 3). The deposited layer of mainly casein micelles has an additional retention effect [4,17]. Thus, the transmission of proteins (whey proteins, monomeric caseins in the serum, possibly small micelles, depending on the pore size distribution of the membranes) is affected [18], with ∆*p_TM_* as a variable forming a more compact deposited layer as ∆*p_TM_* was raised [20,24]. Therefore, the transmission of caseins and whey proteins decreased with increasing transmembrane pressure (Figure 3). At the lowest transmembrane pressure, the transmission of both protein fractions, casein, and whey protein was the highest for all module types. Moreover, in this context, the undesired casein transmission also decreased by increasing ∆*p_TM_*. The whey protein transmission at a wall shear stress of 76 Pa and 127 Pa in the CTM does not differ significantly (*p* ≤ 0.1). The same applies to HFM. Thus, the transmission is strongly affected by the ∆*p_TM_* and less by the wall shear stress. Although a high wall shear stress is expected to result in a more effective deposit removal, the deposited casein micelles form a more compact, gel-like structure at high ∆*p_TM_* [24], so that significantly higher values of whey protein transmission do not occur. As a consequence, the different protein transmissions can be attributed to the different module designs and, additionally, to the membrane material.

From Figure 3, it becomes evident that the HFM achieves the lowest casein transmission level. At ∆*p_TM_* above 1.0 bar, SWM had the highest whey protein transmission, but, at the same time, the casein concentration in the permeate was also high when compared with that of CTM and HFM. The casein transmission of 7% at ∆*p_TM_* of 0.75 bar is in accordance with data reported in the literature [10,11]. The casein retention in SWM is dependent on the deposit layer formation. SWM permeate contained a significantly higher amount of casein, as reported by Zulewska et al. [10]. At higher ∆*p_TM_* values, which are more common in the dairy industry, the casein transmission is reduced. The difference in casein transmission between the different membranes may be due to the different pore size distributions of the membranes, although the nominal pore size was specified as 0.1 µm for all three membranes. Furthermore, the higher casein transmission can be explained by the fact that the SWM was operated at the maximum of the recommended axial flow velocity [39] and, therefore, deposit formation was at its lowest possible level. Additionally, SWM are affected by an inhomogeneous deposit layer formation on the membrane surface caused by extreme differences in shear stress, depending on the position within the spacer network [26]. Therefore, the protein transmission can be expected to be higher in areas of high shear, which leads to a higher overall transmission, when compared to CTM and HFM. Casein and whey protein transmission are both affected by the inhomogeneous deposit layer formation. However, this has a stronger effect on casein transmission since caseins are present as micelles and they are preferably retained by the deposited layer. The deposit layer formations in CTM and HFM are more homogeneous when compared to SWM [19].

### 3.3. Impact of the Filtration Module on the Fractionation Efficiency

**Figure 4:**
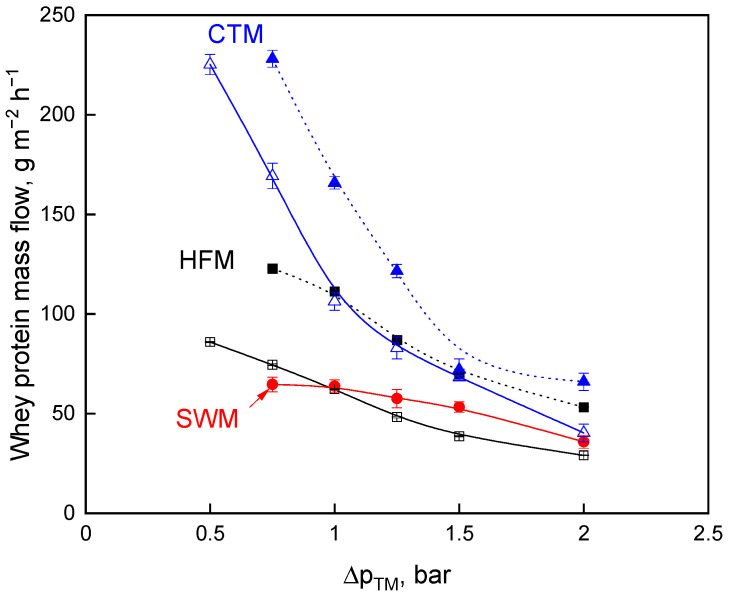
Mass flow of whey proteins as a function of transmembrane pressure, ∆*p_TM_*, for skim milk microfiltration for spiral wound membrane (SWM; •), ceramic tubular membrane (CTM; △ *τ_w_* = 76 Pa; ▲ *τ_w_* = 127 Pa), and hollow fiber membrane (HFM; □ *τ_w_* = 23 Pa; ■ *τ_w_* = 48 Pa).

Higher crossflow velocities going along with higher pressure drops along the module for CTM and HFM lead to higher flux values (Figure 2), but also to an increase of the minimally adjustable ∆*p_TM_*, whereby whey protein transmission is reduced (Figure 3). Hence, the open question is whether the comparison of the membranes with constant feed volume flow or with constant pressure drop yield a higher mass flow. The dependence of flux and protein transmission on the ∆*p_TM_* results in a ∆*p_TM_* optimum for the milk protein fractionation. Being equivalent to the mass flow of the aqueous phase, i.e., flux, the whey protein mass flow was calculated according to Equation (9) and plotted against the ∆*p_TM_* (Figure 4). This provides an additional and more suitable perspective on the speed of protein fractionation than flux and protein transmission alone.

At the higher wall shear stress of 127 Pa and 48 Pa for CTM and HFM, higher whey protein mass flows were obtained, respectively. Higher flux values are achieved due to higher crossflow velocities, producing a stronger effect on whey protein mass flow than reaching lower ∆*p_TM_* (0.5 bar as compared to 0.75 bar) and, thus, increased whey protein transmission. The comparison at constant feed volume flow in relation to a comparison at constant pressure drop shows higher mass flow at a constant pressure drop dependent on the ∆*p_TM_*. The maximum of the mass flow was at a ∆*p_TM_* of 0.75 bar and a pressure drop of 1.3 bar·m^−1^ for CTM, HFM, and SWM, respectively (Figure 4). Operating at this point, CTM provides the highest whey protein mass flow of 229 g·m^−2^·h^−1^ flowed by HFM (123 g m^−2^ h^−1^) and SWM (64 g·m^−2^·h^−1^). In other words, CTM provides 1.9 times and 3.6 times more whey protein mass flow when compared to HFM and SWM, respectively. This results from the combination of the high whey protein transmission and the high flux of the CTM at a ∆*p_TM_* of 0.75 bar compared to HFM and SWM.

The casein-to-whey protein ratio in the permeate was calculated at the whey protein mass flow maximum in order to characterize the efficiency of the milk protein fractionation. The casein-to-whey protein ratio for the SWM was 0.77. This is in accordance with the literature, where the casein-to-whey protein ratio in SWM MF permeate was reported as 1.0 after a MF filtration time of 30 min. [40]. The casein-to-whey protein ratio of the HFM was 0.13 and the milk protein fractionation was, therefore, most efficient when compared to that of the SWM and CTM with a casein-to-whey protein ratio of 0.35, which is also in the range of the previously reported data [31]. We assume that the difference in fractionation efficiency between SWM and HFM/CTM results from the fact that the flow properties in SWM were responsible for faster and, in some terms, more intense membrane fouling and the fouling by casein changed the separation characteristics.

### 3.4. Impact of the Packing Density of the Module on the Filtration Efficiency

The flux and the protein mass flow are specific values per square meter membrane installed, as shown in Figure 2 and Figure 4. However, the protein mass flow output per module, which results from the overall membrane area installed per module volume or module volumetric footprint, appears to be a more suitable measure when comparing the module types, given the greatly differing packing densities. The ability to pack a very large membrane area into a single module, depending on fiber diameter, is an advantage of HFM modules [13]. Therefore, modules with approximately the same dimensions, i.e., module diameter, were compared for flux, transmission, and whey protein mass flow in relation to the different module type volumes, as characterized in Table 2. From these data, the whey protein mass flow per module and per volume was calculated and is shown in Table 5 at its ∆*p_TM_* optimum for constant feed volume flow and constant pressure drop.

When HFM and CTM are operated at the same feed volume flow, they perform better in terms of transmission as well as mass flow at their ∆*p_TM_* optima when compared to SWM, although they are compared at low crossflow velocities (Table 5). This shows a possibility to obtain a high output of whey protein mass flow at low volume flow and low transmembrane pressure and, thus, low energy input. Even at the same feed volume flow rate, HFM and CTM are competitive when compared to SWM. This is also shown by the comparison operating at the same pressure drop: in terms of specific flux, transmission, and specific mass flow of whey proteins, CTM provides the best filtration performance. However, CTM have the lowest module volume-specific membrane packing density with 119 m^2^·m^−3^ as compared to HFM with 449 m^2^·m^−3^ and SWM with 723 m^2^·m^−3^ (Table 2). Therefore, the whey protein mass flow per module and per volume in a CTM module is notably lower when compared to the SWM and HFM modules. Viewed from the perspective of the SWM module, a high packing density provides a high whey protein mass flow per module and per volume although the SWM module seems to provide low filtration performance in terms of the lowest membrane area-specific flux and lowest specific whey protein mass flow as compared to HFM and CTM. The flux of CTM was 2.4 times higher and the whey protein mass flow 3.6 times higher than in SWM, but the SWM module provided a 2.7 times higher whey protein mass flow per module than the CTM module. With the whey protein mass flow per module as the main criterion for the performance comparison of membrane modules, it is obvious that the HFM module provided the best filtration performance when compared to SWM and CTM. Although HFM performed at the level of CTM in terms of specific flux, but superior to SWM, the HFM module provided the best whey protein mass flow per module. Taking all aspects into consideration, HFM provided a higher specific filtration area when compared to that of CTM. It can be said that free flow cross-sections of the channels lead to better control of the deposit layer than the spacer-filled flow channels in SWM. Thus, a more efficient milk protein separation with high whey protein mass flow could be achieved.

## 4. Conclusions

The overall economics of the membrane system should be considered when selecting a suitable membrane module for milk protein fractionation. Here, not only the different acquisition, manufacturing, and maintenance costs play a decisive role. In addition, the effect of the selection of process parameters on the operating costs as well as the module dimension (membrane diameter and length). Ceramic membranes induce higher manufacturing costs, but they are more durable and resistant against processing and chemical stress than polymer membranes. However, SWM provide acceptable flux values, even at lower feed flow rates when compared to CTM and HFM. When membrane modules are operated at high crossflow velocity, a better control of membrane fouling could be achieved in contrast to operating at the same feed volume flow, however, at the cost of higher energy expenditures to produce higher volume flow rates. This, in turn, also increases the effect of backpressure. The results of the studied membrane systems indicated that the key criterion of a comparative assessment for an effective milk protein fractionation is the packing density and, thus, the footprint of a membrane module. An increase in the cross-section of the modules would lead to an increase in required volume flow to feed the system and an extension of the module length to more intense length-dependent effects of the MF. Both of the effects increase the operating costs during fractionation. The results reported here for SWM, CTM, and HFM, however, confirm that lower transmembrane pressures and high crossflow velocities seem to be appropriate for performing milk protein fractionation with these module types. Based on the data that are reported in this study, HFM, which are less often applied in the dairy industry for milk protein fractionation, appear to be a suitable alternative to the more established SWM or CTM, which would be interesting to see verified at the industrial level. The advantage of HFM could become even higher compared to the results presented here, depending on actual and future HFM developments regarding maximization of membrane packing density.

## Figures and Tables

**Table 1:** The properties of water, milk, and permeate at a temperature of 55 °C.

Properties	55 °C
*η_Water_* [10^−6^·kg·m^−1^·s^−1^]	504.2 ± 8.7
*η_Milk_* [10^−6^·kg·m^−1^·s^−1^]	922.1 ± 11.3
*η_Permeate_* [10^−6^·kg·m^−1^·s^−1^]	730.8 ± 5.9
*φ_Water_* [kg·m^−3^]	985.7 ± 1.6
*φ_Milk_* [kg·m^−3^]	1022.5 ± 4.7
*φ_Permeate_* [kg·m^−3^]	1009.4 ± 2.1

**Table 2:** Module dimensions of hollow fiber membrane (HFM), spiral wound membrane (SWM), and ceramic tubular membrane (CTM).

Type	Module Length, m	Module Diameter, m	Membrane Surface, m^2^	Module Volume, L	Packing Density, m^2^·m^−3^
HFM	1.02	0.16	9.3	20.7	449
SWM	0.97	0.17	16.4	22.6	723
CTM	1.02	0.13	1.7	14.4	119

**Table 3:** Mean crossflow velocity, *v*, the resulting pressure drop, ∆*p_L_*, the wall shear stress, *τ_w_*, and the wall shear rate, *γ_T_*, for spiral wound membrane (SWM), ceramic tubular membrane (CTM), and hollow fiber membrane (HFM) at a feed volume flow of $\dot{V}$ = 20 m^3^·h^−1^.

Type	Mean Crossflow Velocity *v*, m·s^−1^	Pressure Drop ∆*p_L_*, bar·m^−1^	Wall Shear Stress *τ_w_*, Pa	Wall Shear Rate *γ_T_*, 10^3^·s^−1^
HFM	1.6 ± 0.01	0.6 ± 0.01	23 ± 2.1	25 ± 2.1
SWM	0.6 ± 0.01	1.3 ± 0.03	-	-
CTM	3.3 ± 0.01	0.8 ± 0.04	76 ± 8.4	82 ± 10.2

**Table 4:** Mean crossflow velocity, *v*, the resulting pressure drop, ∆*p_L_*, the wall shear stress, *τ_w_*, and the wall shear rate, *γ_T_,* for spiral wound membrane (SWM), ceramic tubular membrane (CTM), and hollow fiber membrane (HFM) at a pressure drop of ∆*p_L_* = 1.3 bar·m^−1^.

Type	Mean Crossflow Velocity *v*, m·s^−1^	Pressure Drop ∆*p_L_*, bar·m^−1^	Wall Shear Stress *τ_w_*, Pa	Wall Shear Rate *γ_T_*, 10^3^·s^−1^
HFM	3.2 ± 0.01	1.3 ± 0.01	48 ± 2.1	52 ± 2.9
SWM	0.6 ± 0.01	1.3 ± 0.03	-	-
CTM	4.7 ± 0.01	1.3 ± 0.04	127 ± 8.4	138 ± 9.5

**Table 5:** Filtration performance of hollow fiber membranes (HFM), ceramic tubular membranes (CTM), and spiral wound membranes (SWM) for skim milk microfiltration at their ∆*p_TM_* optima.

Module	HFM	CTM	SWM	HFM	CTM	SWM
	$\dot{V}$ = 20 m^3^·h^−1^			∆*p_L_* = 1.3 bar·m^−1^		
∆*p_TM_* optimum, bar	0.5	0.5	0.75	0.75	0.75	0.75
Flux, L·m^−2^·h^−1^	33	67	32	55	78	32
Whey protein transmission, %	50	67	46	44	51	46
Whey protein mass flow, g·m^−2^·h^−1^	86	225	64	123	229	64
Whey protein mass flow per module, g·h^−1^	800	384	1050	1144	389	1050
Whey protein mass flow per volume, g·m^−3^·h^−1^	38,614	26,775	46,272	55,227	27,251	46,272
